# Supplementary figures and images for: The calcitron: A simple neuron model that implements many learning rules via the calcium control hypothesis
Source: PLoS Comput Biol. 2025 Jan 29;21(1):e1012754. doi: 10.1371/journal.pcbi.1012754 (PMC11835382; doi:10.1371/journal.pcbi.1012754)

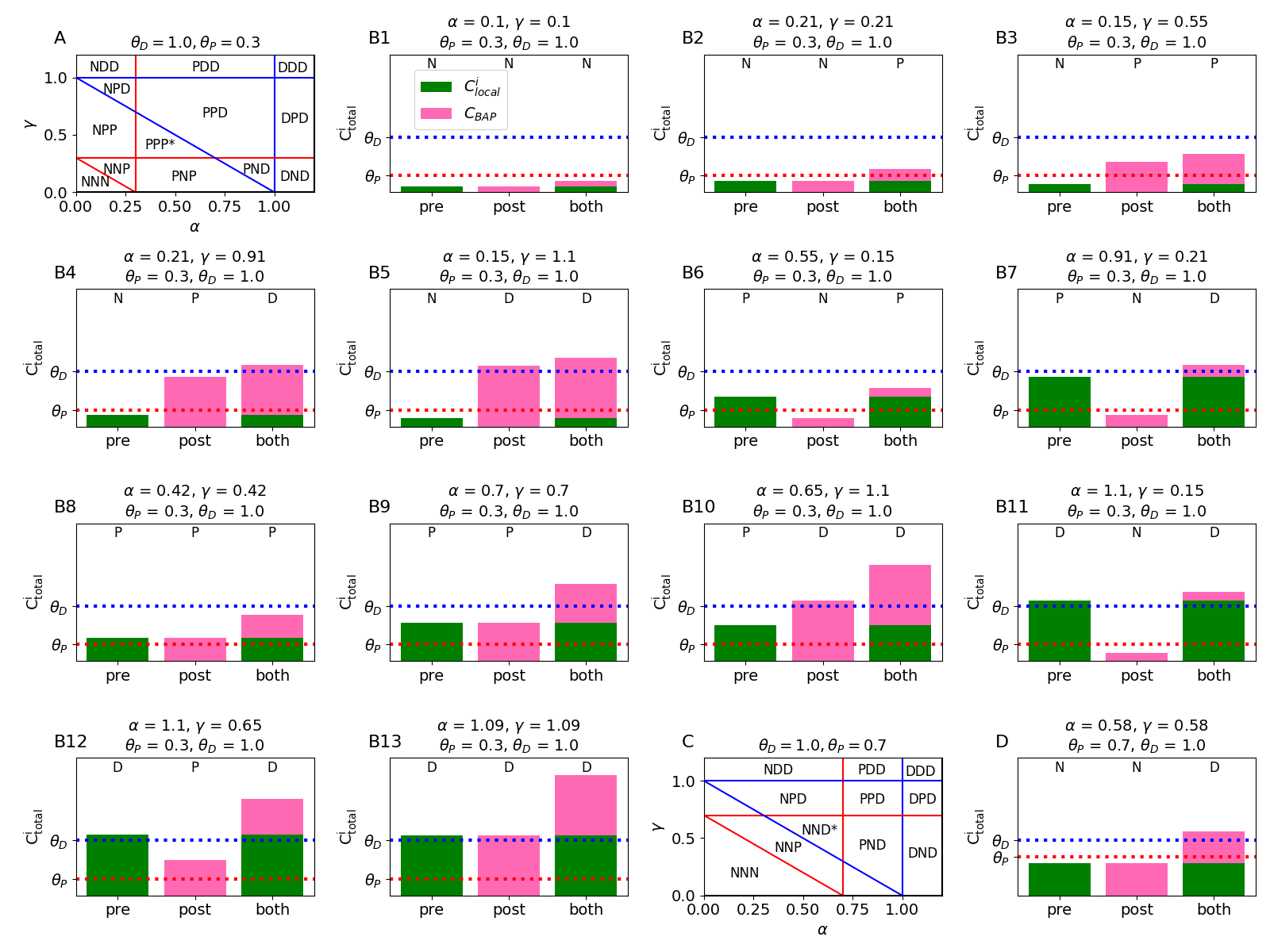

Supplement: S1 Fig — (A) First scenario for reversed calcium thresholds (i.e., θP<θDwhere the potentiative region is larger than the pre-potentiative region, i.e., θD−θP>θP. Asterisk indicates rule (PPP) that can’t be implemented under the second threshold scenario from (C). (B1–B13) Each of the 13 regions from panel (A) represented as a bar plot. (C) Second calcium-threshold scenario, where the pre-potentiative region is larger than the potentiative region, i.e., θP>θD−θP. Asterisk indicates rule (NND) that can’t be implemented under the first threshold scenario from (A). (D) Bar plot for the NND rule from panel (C). (TIFF) [file pcbi.1012754.s001.tiff]
